# Self-Regulation Mediates Therapeutic Horseback Riding Social Functioning Outcomes in Youth With Autism Spectrum Disorder

**DOI:** 10.3389/fped.2022.884054

**Published:** 2022-06-28

**Authors:** B. Caitlin Peters, Zhaoxing Pan, Hannah Christensen, Robin L. Gabriels

**Affiliations:** ^1^Temple Grandin Equine Center, Department of Animal Sciences, Colorado State University, Fort Collins, CO, United States; ^2^Department of Pediatrics, School of Medicine, University of Colorado Denver, Denver, CO, United States; ^3^Department of Psychiatry, School of Medicine, University of Colorado Denver, Denver, CO, United States

**Keywords:** equine-assisted services, therapeutic horseback riding, autism spectrum disorder, mediation analysis, human-animal interaction, self-regulation, social functioning

## Abstract

Emerging evidence suggests therapeutic horseback riding improves self-regulation behaviors, social functioning, and language in youth with autism spectrum disorder (ASD). It has been theorized that interacting with horses is calming for youth with ASD, which may influence social and language outcomes. The current study is an exploratory secondary mediation analysis of a previously published randomized controlled trial of therapeutic horseback riding for youth with ASD. We hypothesized that self-regulation would mediate therapeutic horseback riding's effect on social and language outcomes in youth with ASD. Results indicate that self-regulation mediates therapeutic horseback riding's effect on social, but not language outcomes. This paper provides support for the hypothesis that interacting with horses may have a calming effect that serves as a platform for improving social outcomes in youth with autism.

## Introduction

Autism spectrum disorder (ASD) is defined by restricted, repetitive behaviors, and difficulties with social communication and interaction abilities (1). Individuals with ASD are neurodiverse, exhibiting unique strengths such as strong memory skills or visuospatial skills (2, 3) as well as limitations in social skills that persist throughout the lifespan, and can be associated with detrimental outcomes such as social isolation and unemployment (4, 5). The Diagnostic and Statistical Manual Version 5 highlights specific social-emotional challenges involving social approach, engagement and understanding in individuals with ASD, all of which can increase their risk for emotional dysregulation (1). A majority of youth with ASD have co-occurring psychiatric disorders that include difficulties with self-regulation abilities (6–8), thus highlighting the need for effective interventions for this population.

Impaired self-regulation is believed to be inherent in ASD, as evidenced by difficulty managing emotions (9), heightened physiological arousal/reactivity to daily activities (10, 11), and increased problematic behaviors such as irritability, hyperactivity, aggression, elopement, and self-injury (12–14). Youth with ASD have also demonstrated increased emotional-related internalizing and externalizing behavior problems (e.g., anxiety, depression, aggression, rule-breaking) (15). Youth with ASD tend to be less effective using self-regulation strategies to manage challenges of daily living, compared to typically developing peers (9). Impaired self-regulation in youth with ASD can result in symptoms of anxiety (16), poor social adjustment (17), and poor academic performance (18).

The Yerkes-Dodson theory (19) as interpreted by modern scholars suggests that an optimal level of physiological arousal enhances performance; arousal levels that are too low (e.g., bored, tired) or too high (e.g., anxious, irritable) result in decreased performance (20). Applying this theory to youth with ASD may help explain why their tendency for having high levels of arousal (9) (e.g., irritability and hyperactivity) could further impair their functional use of social and language capacities. Given this theory, interventions targeting self-regulation in individuals with ASD may also improve secondary outcomes such as social and language functioning.

Emerging evidence suggests animals, particularly therapeutic horseback riding (THR), can improve self-regulation related behaviors (i.e., irritability and hyperactivity) in youth with ASD (21). Reviews have also reported that a variety of animal-assisted interventions (AAI) for youth with ASD have found decreased problematic behaviors, increased positive emotions, and decreased physiological and behavioral indicators of stress (22, 23). Given these emerging findings paired with the Yerkes-Dodson theory of optimal arousal leading to enhanced performance, interacting with animals may induce a calm and regulated, yet still motivated and alert state of arousal in youth with ASD.

Gabriels et al. (21) completed the largest randomized controlled trial of THR for youth with ASD to date. THR involves teaching horsemanship skills to individuals with special needs and allows for individuals to interact and work as a team with their horse in a structured setting through both ground-based (i.e., tacking, grooming) and mounted activities. Gabriels et al. (21) demonstrated that 10-weeks of 1-h small group THR lessons significantly improved self-regulation (i.e., irritability and hyperactivity), social functioning (i.e., social communication and social cognition) and language (i.e., number of words and new words) in youth with ASD ages 6 to 16 years. For the current study, we conducted a secondary exploratory analysis of archived raw data from the Gabriels et al. (21) study to test the hypothesis that THR's effects on self-regulation (i.e., decreased irritability and hyperactivity) may explain this intervention's effect on the observed social and language outcomes.

While randomized controlled trials can answer questions related to efficacy, they do not answer questions regarding intervention *mechanisms*, defined as “the basis for the effect, i.e., the processes or events that are responsible for the change; the reasons why change occurred or how the change came about” (p. 3) (24). Mediation analyses embedded within a randomized controlled trial can be used to investigate the causal mechanisms by which an intervention affects specific outcomes. To do so, mediation analyses separate the total effect of an intervention into an indirect effect and a direct effect (25). The indirect effect represents the effect of the intervention that operates through the mediator, in this case self-regulation behaviors (irritability and hyperactivity). The remaining direct effect then represents the effect of the intervention that operates through all other mechanisms, excluding the mediator. A significant indirect effect suggests that the selected mediator acts a *mechanism*, helping to explain *how* the intervention achieves designated outcomes. Clearer understanding of causal mechanisms can help guide the AAI field by (1) optimizing manualized interventions to target the change process to meet individual needs and (2) identifying who may benefit most from an AAI intervention. To date, no known mediation analyses have been published that examine potential mechanisms of THR's benefits for youth with ASD.

Therefore, the purpose of this exploratory secondary analysis was to test if self-regulation (defined as irritability and hyperactivity behaviors) mediates THR's effect on social and language outcomes in youth with ASD ages 6 to 16 years. Our primary hypothesis was that pre-post intervention changes in self-regulation would mediate THR's effect on social functioning outcomes (social communication and social cognition). Our secondary hypothesis was that pre-post intervention changes in self-regulation would mediate THR's effect on language outcomes (number of words and new words spoken during a language sample).

## Materials and Methods

The current paper is an exploratory secondary analysis of Gabriels et al. (21); see that randomized controlled trial for an in-depth description of methods summarized here. One hundred and twenty-seven participants with a study-confirmed ASD diagnosis were randomized by non-verbal IQ standard scores (≤85 or >85) to one of two 10-week study groups: THR intervention or barn activity (BA) control group without horses. Eligibility criteria consisted of participants meeting the Leiter-R Non-Verbal IQ cut-off of ≥40 (26), the Social Communication Questionnaire ASD screening cut-off of ≥15 (27), the Autism Diagnostic Observation Schedule, Second Edition cut-off for ASD (28), and having combined Irritability and Stereotypy subscale score of ≥11 on the Aberrant Behavior Checklist-Community (ABC-C) (29).

### Interventions

#### THR Intervention

In the Gabriels et al. (21) study from which this current paper conducted secondary analyses, participants engaged in 1-h small group weekly lessons led by a PATH, Intl (30) registered therapeutic horseback riding instructor and assisted by volunteers to learn about horses, horse care, and riding skills. Information taught followed a sequence of skills and information outlined in the study intervention manual (31).

#### BA Control Intervention

Similar to the THR group, the participants from the Gabriels et al. (21) study engaged in 1-h small group weekly lessons led by a PATH, Intl registered therapeutic horseback riding instructor and assisted by volunteers to learn about horses and horse care in the sequence outlined in the manual (31). However, this group had no interaction with horses, rather, a life-sized toy horse was used to teach participants skills.

### Behavioral Outcome Measures

Two behavioral outcome measures collected in the Gabriels et al. (21) study were used as outcome variables in the current mediation analyses. First, an unblinded consistent caregiver completed the Social Responsiveness Scale (SRS) (32) before and after the interventions (i.e., 1-month pre-intervention and 1-month post-intervention). The SRS is a 65-item questionnaire measuring social impairments (e.g., social communication and social cognition) in children with ASD. Additionally, a speech therapist blinded to participant's randomization status conducted a 5-min language sample using the Systematic Analysis of Language Transcripts (SALT) (33) to measure number of words and new words spoken by each participant before and after the interventions (i.e., 1-month pre-intervention and 1-month post-intervention).

The current mediation analyses also used the ABC-C data collected by Gabriels et al. (21) as the mediating variable. In the Gabriels et al. (21) study the ABC-C was completed by a consistent caregiver before and after the interventions, as well as weekly during the 10-week interventions. The ABC-C is a 58-item symptom checklist that measures the presence and severity of problem behaviors in children and adults with developmental disabilities in the community. Test-retest reliability of ABC-C parent-ratings range from *r* = 0.80–0.95, demonstrating scores are stable in the absence of intervention (32). The ABC-C is frequently used in ASD clinical trials and has concurrent validity with measures of behavior (34, 35). Given that the Irritability and Hyperactivity subscales demonstrated significant improvement in the Gabriels et al. (21) study and were highly correlated with one another (baseline *r* = 0.64, *p* < 0.001; end-of-treatment *r* = 0.73, *p* < 0.001; difference *r* = 0.75, *p* < 0.001), to test our hypotheses we created a combined variable named “self-regulation” by calculating the sum of the ABC-C Irritability and Hyperactivity scores.

### Description of Analysis Data Set

Eighty-nine participants (THR *n* = 45 and BA control *n* = 44) who followed the Gabriels et al. (21) research protocol with complete ABC-C and SRS measures assessed 1-month pre- and post-intervention are included in this exploratory secondary analysis. Of note, one THR and one BA control participant did not have the SALT evaluation at post-intervention, resulting in a sample size of 87 for the analyses involving the SALT. For the sensitivity analyses, all THR participants completed the ABC-C at week 6, but 4 participants in the BA control group did not complete the ABC-C at week 6 so week 7 ABC-C data was used instead (THR *n* = 45; BA *n* = 44).

### Statistical Analysis

Baseline characteristics of participants are summarized using mean and standard deviation for continuous variables and percent distribution for categorical variables. Student *t*-tests for two independent samples were used to assess the efficacy of the THR intervention as compared to the BA control group using post-intervention change scores as the outcomes. In the mediation analyses, the post-intervention change score from baseline serves as the mediator (ABC-C) and outcome variables (SRS, SALT). For ease of interpretation, the change scores for all variables were calculated so that positive scores imply improvement. Therefore, change scores were calculated as baseline value minus post-intervention value for ABC-C and SRS and as post-intervention minus baseline for SALT. Inferences on mediation effects were based on the 95% confidence interval of the product of coefficients of an indirect path (36, 37). The confidence interval was estimated based on 10,000 bootstrap samples. Mplus 7.4 (38) was used for mediation analyses and SAS 9.4 (39) for other analyses. *P* value < 0.05 was deemed to be significant. We completed additional sensitivity analyses using the same mediation model by using the week 6 (or week 7 for participants with missing week 6 data) ABC-C data as the mediating variable.

## Results

Forty-five THR and 44 BA participants were included in the final analyses. Table 1 presents baseline demographic and clinical characteristics. Groups did not significantly differ at baseline in any of the demographic or clinical variables.

**Table 1:** Participant characteristics.

**Characteristic**	**BA Mean** ***N*** **= 44**	**THR Mean *N* = 45**	***p*** Value^*^
Age (SD)	10.4 (3.0)	10.1 (2.8)	0.62
Sex M/F	40/4	37/8	0.23
Nonverbal IQ (SD)	88.5 (26.9)	85.5 (21.3)	0.60
Race (*N* = 86)			0.38
American Indian/Alaskan Native	3	0	
Asian	2	1	
Black	0	1	
Hawaiian/Pacific Islander	1	0	
White	32	37	
Multiracial	1	2	
Other	3	1	
Ethnicity (Hispanic; *N* = 88)	9	7	0.51
Repetitive Behavior Scale Total	35.0 (21.7)	38.5(19.7)	0.42
Current Seizure Disorder	1	1	0.99
ABC-C Irritability (SD)	15.2 (9.1)	16.4 (10.0)	0.57
ABC-C Hyperactivity (SD)	21.3 (10.2)	21.7 (9.4)	0.86
SRS Social Cognition (SD)	19.6 (5.3)	19.9 (5.0)	0.75
SRS Social Communication (SD)	35.8 (9.7)	35.3 (7.9)	0.78
SALT number of words (SD; *N* = 87)	215.8 (137.4)	275.8 (178.1)	0.27
SALT number of new words (SD; *N* = 87)	102.6 (60.0)	117.5 (66.1)	0.08

*BA, Barn Activity Group; THR, Therapeutic Horseback Riding Group; ADOS-2, Autism Diagnostic Observation Scale, Second Edition; ABC-C, Aberrant Behavior Checklist, Community; SRS, Social Responsiveness Scale; VABS, Vineland Adaptive Behavior Scale; SALT, Systematic Analysis of Language Transcripts,*

* *p Values represent values for t-tests for continuous variables and Chi-square tests for categorical variables*.

### Intervention Effects

Table 2 provides efficacy analyses for the subset of completers included in the current paper. THR participants included in the current analyses demonstrated significantly greater improvements compared to BA control group in self-regulation (*p* = 0.02); SRS social communication (*p* = 0.02); and number of words spoken (*p* = 0.006), and number of new words spoken (*p* = 0.006). This confirms the efficacy analysis reported in Gabriels et al. (21). However, the subset of completers included in this exploratory secondary analysis no longer demonstrated significantly greater improvement in social cognition (*p* = 0.20) compared to the BA control group.

**Table 2:** Efficacy analyses of the subset of completers included in mediation analyses.

**Outcome**	**BA Mean (SD)**	**THR Mean (SD)**	***t*** **(DF)**	***p*** Value^*^
Δ Self-regulation	5.89 (15.08)	13.40 (14.34)	2.41 (87)	**0.018**
Δ SRS Social Communication	1.66 (7.75)	5.60 (7.70)	2.41 (87)	**0.018**
Δ SRS Social Cognition	0.89 (4.62)	2.07 (4.07)	1.28 (87)	0.204
Δ SALT # of words	−10.19 (101.20)	46.77 (88.37)	2.8 (85)	**0.006**
Δ SALT # of new words	−2.72 (34.28)	17.43 (32.13)	2.83 (85)	**0.006**

*ABC-C, Aberrant Behavior Checklist, Community; SRS, Social Responsiveness Scale; SALT, Systematic Analysis of Language Transcripts*.

* *Student t-test for two independent samples. Bold values indicate p < 0.05*.

### Self-Regulation Mediation Effects

**Figure 1:**
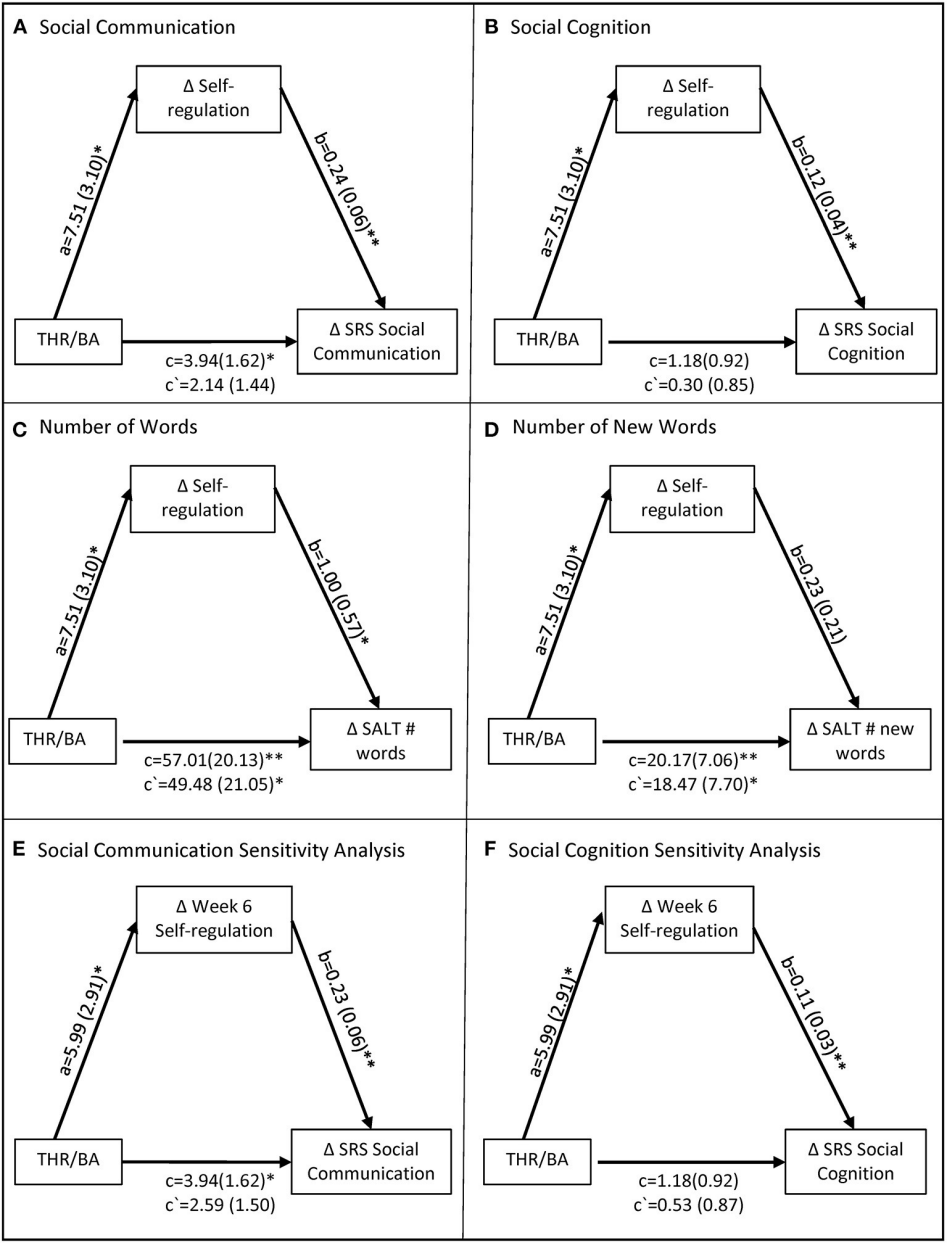
Mediation models of THR's effect on social and language outcomes through self-regulation. Models depict the total effect c, direct effect c', intervention mediator effect a, and the mediator-outcome effect b. Mediation inferences were determined by the confidence interval of the indirect effect ab which is provided in text, not pictured here. Models **(A–D)** depict the primary analyses and models **(E,F)** depict the sensitivity analyses. *BA*, Barn Activity Group; *THR*, Therapeutic Horseback Riding Group; *SRS*, Social Responsiveness Scale; *SALT*, Systematic Analysis of Language Transcripts. *indicates *p* < 0.05. **indicates *p* < 0.01.

Figure 1 illustrates all mediation analyses and provides values for all a, b, c, and c′ paths.

#### Social Communication

Treatment condition had a significant total effect on social communication, such that the THR participants demonstrated significantly greater improvements in social communication [c = 3.94 (1.62), 95% CI = 0.72, 7.09]. The indirect effect of self-regulation on social communication was significant, as demonstrated by a confidence interval that did not contain zero [ab = 1.80 (0.86), 95% CI = 0.29, 3.69]. Thus, self-regulation was a significant mediator such that the THR treatment condition was related to improved self-regulation [a = 7.51 (3.10), *p* = 0.02], which in turn was related to improved social communication [b = 0.24 (0.06), *p* = 0.000].

#### Social Cognition

Treatment condition did not have a significant total effect on social cognition [c = 1.18 (0.92), 95% CI = −0.62, 2.95]. Despite the absence of a total effect, the indirect effect of self-regulation on social cognition was significant [ab = 0.88 (0.46), 95% CI = 0.12, 1.89]. Thus, self-regulation was a significant mediator such that THR treatment condition was related to improved self-regulation [a = 7.51 (3.10), *p* = 0.02], which in turn was related improved social cognition [b = 0.12 (0.04), *p* = 0.001].

#### Number of Words

Treatment condition had a significant total effect on number of words, such that THR participants demonstrated significantly greater improvements in number of words [c = 57.01 (20.13), 95% CI = 17.13, 95.73]. The indirect effect of self-regulation on number of words used was not significant, as indicated by a confidence interval that includes zero [ab = 7.54 (5.87), 95% CI = −0.44, 21.76]. While the THR treatment condition was related to improved self-regulation [a = 7.51 (3.10), *p* = 0.02], improved self-regulation was not significantly related to number of words [b = 1.00 (0.57), *p* = 0.08]. Therefore, these analyses did not support the hypothesis that self-regulation mediated THR's effect on number of words spoken during the language sample.

#### Number of Different Words

Treatment condition had a significant total effect on number of different words, such that THR participants demonstrated significantly greater improvements in number of different words [c = 20.17 (7.06), 95% CI = 6.35, 33.94]. The indirect effect of self-regulation on number of different words was not significant [ab = 1.69 (1.84), 95% CI = −1.07, 6.08]. While THR treatment condition was related to improved self-regulation [a = 7.51 (3.10), *p* = 0.02], improved self-regulation was not significantly related to number of new words [b = 0.23 (0.21), *p* = 0.28]. Therefore, these analyses did not support the hypothesis that self-regulation mediated THR's effect on number of different words spoken during the language sample.

#### Sensitivity Analysis

To examine the robustness of the findings above, we conducted an additional sensitivity analysis using the mid-intervention self-regulation score, previously defined, as the mediator. The subgroup of THR and BA participants included in these analyses demonstrated significant between-group differences in self-regulation change score from baseline by week 6; these differences remained consistent for the remainder of the 10-week intervention. Therefore, we selected week 6 self-regulation score as the mediator for the sensitivity analyses. The indirect effect of week 6 self-regulation on social communication was significant, as indicated by a confidence interval that does not include zero [ab = 1.36 (0.84), 95% CI = 0.05, 3.32]. Similarly, the indirect effect of week 6 self-regulation on social cognition was also significant [ab = 0.65 (0.38), 95% CI = 0.02, 1.50].

## Discussion

The paper reports an exploratory secondary mediation analysis of the Gabriels et al. (21) study. Results support our primary hypothesis that THR participants' improved self-regulation behavior identified for these analyses (i.e., irritability and hyperactivity) mediates THR's effect on their previously observed improvements in social behavior outcomes (i.e., social communication and social cognition). However, results did not support our secondary hypothesis; THR participants' improved self-regulation behaviors did not mediate THR's effect on the number of words and new words spoken during a language sample. This paper also reports a sensitivity analysis using self-regulation behavior collected at week 6 by Gabriels et al. (21) as the mediator. Our analyses revealed that the significant between group (THR compared to BA control) differences in self-regulation behaviors began at week 6, and these week 6 improvements in self-regulation behaviors also mediated the social communication and social cognition outcome variables. These sensitivity analyses support the robustness of self-regulation as a mediator of THR social behavior outcomes. This paper is the first known to report potential mediators of an AAI, specifically with horses, for youth with ASD. The mediation analyses reported in this paper lend support to the widely-held hypothesis that interacting with animals is calming for youth with ASD (40) and suggests that its' positive effect on self-regulation behaviors (i.e., irritability and hyperactivity) may be an important mediating ingredient in the human–equine interaction that can lead to positive changes in social outcomes. Additionally, the sensitivity analyses reported in this paper support the mediation analysis mid-intervention data point defined for our current follow-up randomized controlled trial designed to evaluate the physiological mechanisms of THR in youth with ASD and co-occurring psychiatric diagnoses (5R01HD097693; clinicalTrials.gov Record 07-1148).

### Self-Regulation as a Mediator for THR Social Outcomes

Several studies have found that a variety of AAIs can be calming for youth with ASD. For example, O'Haire et al. (41) found that youth with ASD ages 5–13 demonstrated decreased physiological arousal during play with peers when in the presence of a guinea pig compared to a toy. Similarly, Silva et al. (42) found that children and adults with ASD demonstrated increased heart rate variability, an indicator of the autonomic nervous system and decreased stress, during structured interaction with a dog as opposed to a robotic dog. These studies suggest that interacting with various animals may be calming for individuals with ASD. THR is unique to other AAIs in that participants can physically mount and ride the horse, providing unique vestibular, proprioceptive, and visual stimulation. Multi-sensory interventions often lead to improved behavioral regulation in youth with ASD (43, 44), and thus the unique multisensory experience of riding a horse may further contribute to a calming effect of THR.

The current paper suggests that this calming effect mediates THR's effect on social outcomes. Several previous AAI studies have found concurrent self-regulation and social outcomes in youth with ASD (45, 46). There is increasing scientific interest in assessing self-regulation and social outcomes of THR in youth with ASD (47, 48). However, to our knowledge the current paper is the first to report a mediation analysis, examining improved self-regulation as a *mechanism* for social outcomes within a randomized controlled trial. Consistent with the Yerkes-Dodson theory (19), these analyses suggest that the calmer state of arousal (i.e., decreased irritability and hyperactivity) induced by THR allows for a child's full expression of their social capacities, and perhaps allows a child to better develop age-appropriate social skills.

Identification of improved self-regulation as a likely mechanism of change in THR can guide referring practitioners to make more targeted referrals to THR for youth with ASD who have chronic poor self-regulation. Additionally, riding instructors can also better tailor their services to optimize THR's calming effect. For example, if a participant demonstrates better self-regulation during riding compared to unmounted activities, the instructor may tailor lessons to include more riding activities for that participant in order to optimize THR's direct effect on self-regulation, and indirect effect on social outcomes.

### Self-Regulation Did Not Mediate THR Language Outcomes

The current analyses did not support self-regulation as a mediator of THR language outcomes. More research is needed to better understand the mechanisms by which THR improves language in youth with ASD. For example, perhaps the nature of THR as a group intervention that requires participants to follow verbal directions and use verbal commands such as “walk on” may explain participants' improved language in the previous study.

### Limitations and Future Research

As an exploratory secondary analysis of a previous randomized controlled trial, this study is limited in that mediation was not originally included in Gabriels et al. (21) original study design. Future research can design THR randomized controlled trials to intentionally answer questions of mediation by prospectively powering the study to assess mediators measured at a mid-intervention timepoint (25). Because Gabriels et al. (21) did not prospectively design the randomized controlled trial to include mediation analyses, there were missing week 6 ABC-C datapoints. This study is also limited by the under-representation of females in the sample. Finally, this study is further limited by reliance on parent-report measures for self-regulation and social outcomes; future studies can include physiological indicators of arousal/regulation as well as expand the types of outcome measures to further understand the hypothesized regulating effect of THR.

## Conclusion

This is the first known paper of its kind to support the often-hypothesized theory that AAIs induce a calm-regulated state in youth with ASD, which may mediate improvements in social outcomes. This paper helps move the AAI field beyond efficacy trials to provide beginning evidence to answer more nuanced questions of how THR works for youth with ASD. The historical theoretical focus on AAI leading to arousal regulation/conditioning is an ideal target for mediation analyses via physiological measurements. The knowledge to be gained from additional self-regulation mediation research will provide evidence for self-regulation response patterns as a causal pathway to THR outcomes. This has the potential to guide future researchers who wish to explore mechanisms by which AAI demonstrates benefits beyond THR and to other populations, particularly a variety of mental health populations with poor self-regulation.

## Data Availability Statement

The original contributions presented in the study are included in the article/supplementary materials, further inquiries can be directed to the corresponding author/s.

## Ethics Statement

The studies involving human participants were reviewed and approved by Colorado Multiple Institution Review Board, University of Colorado. Written informed consent to participate in this study was provided by the participants' legal guardian/next of kin.

## Author Contributions

BP conceived of the paper's hypotheses and was primarily responsible for manuscript preparation. ZP conducted all analyses and assisted in manuscript preparation. HC served as study coordinator and assisted in manuscript preparation. RG served as principal investigator on the study, overseeing all data collection and analysis, and assisted in manuscript preparation. All authors approved the final version of the manuscript.

## Funding

This study was supported by grant R01NR012736 from the National Institute of Nursing Research. Article processing charges were supported by Colorado State University Libraries Open Access Research and Scholarship Fund.

## Conflict of Interest

The authors declare that the research was conducted in the absence of any commercial or financial relationships that could be construed as a potential conflict of interest. The reviewer TG declared a shared affiliation with the author BP.

## Publisher's Note

All claims expressed in this article are solely those of the authors and do not necessarily represent those of their affiliated organizations, or those of the publisher, the editors and the reviewers. Any product that may be evaluated in this article, or claim that may be made by its manufacturer, is not guaranteed or endorsed by the publisher.
